# Immunohistochemical analysis to detect a molecular signature in intervertebral disc degeneration

**DOI:** 10.1007/s00418-025-02434-w

**Published:** 2025-11-25

**Authors:** Letizia Penolazzi, Chiara Angelini, Riccardo Nadalini, Anna Chierici, Elisabetta Lambertini, Chiara Sief, Pasquale De Bonis, Roberta Piva

**Affiliations:** 1 Department of Neuroscience and Rehabilitation, University of Ferrara, Ferrara, Italy; 2 Department of Translational Medicine, University of Ferrara, Ferrara, Italy; 3 Laboratorio Centralizzato Di Ricerca Preclinica, University of Ferrara, Ferrara, Italy; 4 Department of Neurosurgery, Sant’Anna University Hospital of Ferrara, Ferrara, Italy

**Keywords:** Intervertebral disc degeneration, Immunohistochemistry, Biochemical markers

## Abstract

Intervertebral disc degeneration (IDD) is known as a primary contributor to low back pain, a debilitating condition which is the leading cause of disability worldwide. Traditionally, its assessment is based on clinical parameters, including magnetic resonance imaging (MRI). However, patients with similar radiological findings may have significantly different prognoses suggesting the involvement of patient-specific biomarkers and little-investigated molecules supporting the complexity of the pathophysiological microenvironment of the intervertebral disc (IVD). We conducted a study on IVD biopsies from 40 patients with mild IDD (Pfirrmann III), to identify a potential molecular signature that correlates with clinical and behavioral parameters including sex, age, smoking, body mass index (BMI), duration of symptoms prior to surgery, inflammatory cell density, or surgical site. Immunohistological analysis focused on the expression of proteins involved in the defense against oxidative stress, in the maintenance of IVD homeostasis, and energy metabolism: the transcription factors FOXO3a, HIF1α, Bry, the enzyme SOD2, and the glucose transporter GLUT1. Significant differences in protein expression were observed only in relation to Pfirrmann grade. Within the grade III subgroup, expression levels did not vary with patient-specific parameters or clinical outcomes such as complete healing, recurrence, or persistent pain after surgery. This highlights the importance of broadening the scope of assessment in pathological conditions such as IDD. Rather than limiting the evaluation to the expression level of a single protein marker, it is crucial to collect comprehensive data on the various factors that may influence individual patient responses to disc degeneration.

**Supplementary Information:**

The online version contains supplementary material available at 10.1007/s00418-025-02434-w.

## Introduction

The intervertebral disc (IVD) is a fibrocartilaginous structure that absorbs mechanical load and provides flexibility to the spine (Hickman et al. 2022). It is frequently affected by inflammation and degeneration, leading to a multifactorial disease known as intervertebral disc degeneration (IDD), which affects approximately 80% of the world’s population and currently has no cure (Hoffeld et al. 2023). IDD often leads to secondary clinical symptoms such as low back pain and sciatica, significantly impacting patients’ quality of life and contributing to increased healthcare costs. Despite its high prevalence, the risk factors and pathogenetic mechanisms of IDD have not been fully explored. Traditionally, IDD assessment is based almost exclusively on clinical parameters, including magnetic resonance imaging (MRI), which provides data to categorize the severity of disc degeneration through the Pfirrmann classification system (Tamagawa et al. 2022). MRI has become an indispensable tool, alongside pain assessment scales, in clinical decision-making, offering a consistent framework to guide diagnosis and treatment planning. However, in light of emerging data, there is a growing need to improve and optimize this approach. Recent literature highlights strong interest, among both clinicians and basic researchers, in improving the diagnosis and prognosis of IDD through the identification of patient-specific and lifestyle-related risk factors that may contribute to the onset and progression of the disease (Hoffeld et al. 2023). At the same time, in recent years, there is increasing interest in a combined approach that integrates MRI with biochemical profiling of systemic markers and molecular analysis of disc tissue. This multimodal strategy aims not only to improve diagnostic accuracy and stratify patients by disease severity but also to better understand the mechanisms underlying IDD and predict therapeutic responses (Francisco et al. 2023; Leite Pereira et al. 2023). In particular, in the context of identifying molecules involved in signaling pathways critical for maintaining disc health, researchers are moving toward a more personalized approach to diagnosis and treatment (Zhou et al. 2024; Radek et al. 2016).

Many questions remain unanswered, including why the prognosis varies so significantly among patients with apparently similar radiological findings (Yang et al. 2023a, b). For instance, some individuals with severe disc degeneration detected by MRI may experience minimal symptoms, while others with mild degeneration experience severe, debilitating pain and functional impairment (Machino et al. 2022). The reasons for this discrepancy remain a matter of debate. It is reasonable to believe that several factors, beyond those commonly considered, such as physiological aging or mechanical overload, deserve further consideration (Freidin et al. 2019). It is well accepted that the individual responses to disc degeneration may depend on genetic predisposition, environmental factors, lifestyle, and comorbidities (Hoffeld et al. 2023). In addition, a molecular signature may be associated with variability in disease progression and treatment response. The search for specific biomarkers, molecular profiles, and risk factors may lead to various types of screening tests, aimed at identifying promising candidates as both tissue and systemic markers of disc damage or degeneration. These include molecules related to collagen and aggrecan metabolism, enzymes that degrade extracellular matrix (ECM), inflammatory mediators, metabolites, lipids, non-coding RNAs, and other regulators of gene expression (Coquelet et al. 2025). This list is expected to continue to grow, along with the identification of molecules capable of acting as “sensors” of alterations in the IVD microenvironment, promoting cellular protection and maintaining homeostasis, ultimately prolonging the patient’s quality of life.

With this in mind, we sought to understand whether the loss or reduction in expression levels of proteins involved in defense against oxidative stress, maintenance of IVD homeostasis, and energy metabolism could be related to clinical and behavioral parameters of a cohort of patients. We focused on the following proteins: the transcription factors Forkhead box O3 (FOXO3a) (Sanese et al. 2019), hypoxia-inducible factor 1-alpha (HIF1α) (Li et al. 2021), and Brachyury (Bry) (Wu et al. 2023), the enzyme superoxide dismutase 2 (SOD2) (Tamagawa et al. 2024), and the glucose transporter type 1 (GLUT1) (Johnston et al. 2023). These five proteins are part of interconnected signaling pathways that can provide insights relevant to our research objectives. We wondered whether “protective” factors, such as not smoking or a normal body weight, could be associated with maintaining adequate expression levels of these proteins or, conversely, whether unhealthy lifestyles could lead to their downregulation. Immunohistological analysis were performed on biopsies from patients with moderate disc degeneration (Pfirrmann III) and correlated with the following parameters: age, sex, anatomical site of surgery, duration of symptoms before surgery, body mass index (BMI), smoking, and area of inflammatory infiltration in the biopsy.

Although our study is preliminary, it nevertheless suggests the importance of reconsidering the utility of current diagnostic practices and emphasizes the urgency of focusing on alternative diagnostic and prognostic strategies. In particular, the identification of sensitive and specific biomarkers is essential to advance the field of personalized medicine in the context of IDD.

## Materials and methods

### Patient recruitment and ethics statement

From January 2022 to December 2023, a total of 55 donors were enrolled under a research protocol approved by the Ethics Committee of the University of Ferrara and S. Anna Hospital (protocol no. 160998; approved 17 November 2016). Patients ranged in age from 24 to 81, with a mean age of 54. The study cohort consisted of 37 men and 18 women. Additional clinical and demographic characteristics are summarized in Table 1. All participants provided written informed consent prior to sample collection. Patients underwent microsurgical posterior discectomy for the treatment of lumbar disc herniations. Human lumbar disc tissue was harvested from the central nucleus of the disc to ensure consistency and to avoid sampling from the anterior or posterior longitudinal ligaments, the annulus fibrosus, or calcified regions. The degree of disc degeneration was assessed preoperatively by MRI based on the Pfirrmann (PF) classification system. Samples were distributed among Pfirrmann grades I–II, III, and IV–V, as detailed in Table 1. Each lumbar disc specimen was immediately stored in sterile saline and processed within 24 h of surgery to ensure tissue integrity and minimize degradation.

**Table 1:** Percentage distribution of enrolled patients based on the reported parameters

Characteristic	Value^a^
Sex	
Female	18 (33)
Male	37 (67)
Age	
Mean (± SD)	54
Min–max	24–81
Pfirrmann grade	
PF I–II	6 (11)
PF III	40 (73)
PF IV–V	9 (16)
Smoke exposure^b^	
Non-smokers [NS]	20 (41)
Former smokers [FS]	10 (20)
Current smokers [CS]	19 (39)
BMI^b^	
Normal weight [NW]	12 (24.5)
Overweight [OW]	21 (42.8)
Obese [OB]	16 (32.7)
Anatomical site	
L2L3	1 (2)
L3L4	15 (27)
L4L5	25 (46)
L5S1	14 (25)
Duration of symptoms before surgery	
≤ 6 months	36 (65)
> 6 months	19 (35)

^a^Data are presented as *n* (%) unless otherwise specified

^b^Missing data reflect information not released by all patients

### Histochemical analysis

Small fragments of each IVD specimen were washed with 1× phosphate-buffered saline (PBS), fixed in 4% buffered paraformaldehyde for 24 h at 4 °C, embedded in paraffin, and cross-sectioned (5 µm thick). Histochemical sections were deparaffinized, rehydrated, and heated in sodium citrate (pH 6) for antigen retrieval. Slides were then processed with 3% H_2_O_2_ in PBS 1× for 5 min and with blocking solution (PBS 1×/1% bovine serum albumin [BSA]/10% fetal calf serum [FCS]) for 30 min at room temperature (RT). For immunohistochemical evaluation, sections were incubated overnight (4 °C) with a primary antibody against FOXO3a (#ab70315, rabbit anti-human, 1:100 dilution; Abcam, Cambridge, UK), SOD2 (#sc-133134, mouse anti-human, 1:100 dilution; Santa Cruz biotech., Dallas, USA), HIF1α (clone H1alpha67, mouse anti-human, 1:200 dilution; Novusbio, Centennial, USA), GLUT1 (#TA301678, mouse anti-human, 1:200 dilution; Origene, Rockville, USA), and BRY (#ab209665, rabbit anti-human, 1:500 dilution; Abcam), followed by treatment with Vectastain ABC solution (#MP-7500; Vectorlabs, Burlingame, USA) for 30 min. To achieve specificity, the working concentrations of primary antibodies were selected by following the dilution guidelines and technical specifications provided by the antibody’s manufacturer. Reactions were developed using 3,3′-diaminobenzidine (DAB) solution (Vectorlabs), sections were counterstained with hematoxylin, mounted in glycerol and observed under a bright-field microscope (Nikon Eclipse 50i; objectives: Nikon CFI Achr Adl 10× Ph1 Na 0.25 #MRP40102, Nikon CFI Achr Adl 40× Ph1 Na 0.55 #MRP46402; Nikon Corporation, Tokyo, Japan).

### Image analysis

For immunohistochemistry, images were acquired using a CMOS camera with an IMX485 image sensor (Sony, Tokyo, Japan), 8.3-megapixel with a native resolution of 3840 × 2160 pixels. The stained sections were quantified by a computerized video camera-based image analysis system (NIH; US ImageJ software 1.54p version, public domain available at http://rsb.info.nih.gov/nih-image/) under bright-field microscopy (NikonEclipse 50i; Nikon Corporation). A standardized threshold was applied for all analyzed proteins. The number of positive cells in a defined area was counted by two independent observers (double-blind) to ensure objectivity, and the results were expressed as % of positive cells in that area (10 random fields per replicate, five sections per sample).

### Statistical and power analysis

Statistical analyses were performed using GraphPad version 8.0 software (Dotmatics). Statistical differences were determined using Mann–Whitney* U* test or one-way analysis of variance (ANOVA) followed by Tukey’s post hoc test for multiple comparisons. Data distribution was tested for normality using the Shapiro–Wilk test, to determine the appropriate statistical methods for subsequent analyses using Jamovi software version 2.3 (https://www.jamovi.org). To assess the relationships between the expression levels of the analyzed proteins, a correlation matrix was calculated using the Pearson correlation coefficient (FOXO3a, SOD2, HIF1α, GLUT1) and the Spearman correlation coefficient (Bry), based on the data distribution. For grade III PF group a post hoc power analysis was performed to assess the sensitivity of the study based on a total sample size of 40 participants. For correlation analyses, the statistical power to detect Pearson correlation of different effect sizes, small (*r* = 0.10), medium (*r* = 0.30), and large (*r* = 0.50), was approximately 0.094, 0.47, and 0.92, respectively. For both two-group ANOVA and three-group ANOVA with unequal sample sizes, statistical power was assessed considering small, medium, and large effect sizes (*f* = 0.10, 0.25, and 0.40, respectively). For the two-group ANOVA, the resulting power values were approximately 0.095, 0.34, and 0.69, respectively. For the provided parameters (sex, duration of symptoms before surgery, and infiltration area) the observed effect sizes (*f*) ranged from 0.08 to 0.16 for FOXO3a, 0.01–0.05 for SOD2, 0.01–0.06 for HIF1α, 0.08–0.18 for GLUT1, and 0.01–0.25 for Bry. In the case of three-group ANOVA, the resulting power values were approximately 0.079 (small), 0.25 (medium), and 0.58 (large effect size), highlighting limited sensitivity unless the effect was large. For the provided parameters (age, smoking, BMI, anatomical site of surgery) the observed effect sizes (*f*) ranged from 0.12 to 0.38 for FOXO3a, 0.11–0.42 for SOD2, 0.08–0.32 for HIF1α, 0.07–0.36 for GLUT1, and 0.12–0.40 for Bry. The Mann–Whitney *U* test was applied to data relating to postsurgical recovery (presence or absence of chronic pain after discectomy or occurrence of recurrence). Considering the number of patients (*n* = 24), the power values for all analyzed proteins were approximately 0.09 (small), 0.17 (medium), and 0.28 (large). The reference for the Mann–Whitney *U* test was evaluated as < 0.3 small, 0.3–0.5 medium, and > 0.5 large. These estimates were obtained using the free statistical software G*Power. A *p* < 0.05 was considered to indicate a statistically significant difference.

## Results and discussion

Regarding general patient characteristics, this study included 55 patients, 37 men and 18 women, with a mean age of 54 years (24 min, 81 max). As reported in Table 1, herniated discs occurred in 2% of the lumbar vertebrae L2–L3, 27% of the L3–L4, 46% of the L4–L5, and 25% of the lumbosacral joint, the L5–S1 spinal motion segment. The degree of disc degeneration was independently assessed by two experienced spinal surgeons using MRI, assigning each patient a Pfirrmann grade. Most patients (65%) underwent surgery within 6 months of symptom onset, while the remaining 35% were treated after more than 6 months. The median height was 1.75 m (IQR 1.5–1.91) and weight was 80 kg (IQR 54–120). The BMI was 27 kg/m^2^ (IQR 20–41). Of the enrolled patients, 24.5% were of normal weight, while 75.5% were classified as overweight or obese. There are limited in vivo data quantifying the impact of obesity on IVD homeostasis, particularly in terms of mechanical function during daily activities (Segar et al. 2024; Wearing et al. 2006). It is undeniable that increased mechanical load on the spinal disc due to overweight and obesity generates mechanical stress that contributes to the development of IDD (Zhou et al. 2021). However, insufficient data are available on key obesity-related factors, such as lipid metabolism disorders and dysregulation of pro-inflammatory cytokines. Several studies, however, have shown that these factors influence the regulation of cellular phenotype, ECM metabolism, and inflammation in IDD (Curic 2020). Some useful information regarding these parameters can be derived from studies such as the one presented here. We observed a clear correlation between disease incidence and BMI, identifying a BMI greater than 25 as a potential risk factor. Another risk factor monitored was smoking. Of the enrolled patients, 41% were current smokers, 25.6% were former smokers, and 33.3% had never smoked. In total, 32 out of 55 patients had been exposed to smoking during their lifetime. Although smoking is recognized as a contributing factor to the development of several chronic diseases, including IDD, as a result of its influence on bone mineral content, local blood supply, and nutrient metabolism, robust clinical evidence on its specific mechanisms in IDD remains limited (Rajesh et al. 2022). Other parameters collected in the survey were excluded because of the difficulty in relating them to objective values. These include type of pain, work-related fatigue, dietary habits, general musculoskeletal health, and physical activity (e.g., frequency, intensity, regularity, and type). Instead, we present the percentage distribution of comorbidities across Pfirrmann grade groups in Table 2. The analysis revealed that cardiovascular disease (CVD) had the highest prevalence across groups. This result was expected, considering the patients’ mean age of 54 years and an obesity rate of over 70%.

**Table 2:** Percentage distribution of comorbidities among Pfirrmann grade groups

Comorbidities	MetS	CVD	CA	MSD	COPD	GI	MDD
PF I–II	–	6	–	–	–	–	–
PF III	10	30	2.5	27.5	5	7.5	2.5
PF IV–V	9	18	9	18	–	–	–

*MetS* metabolic disease, *CVD* cardiovascular disease, *CA* neoplastic disease, *MSD* musculoskeletal disease, *COPD* chronic obstructive pulmonary disease, *GI* gastrointestinal disease, MDD depressive disorder

**Fig. 1:**
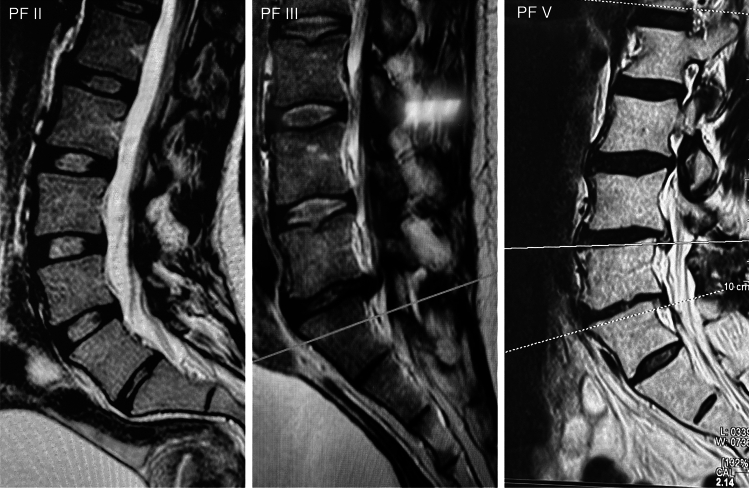
Representative sagittal T2-weighted MR images of IVDs from patients with different Pfirrmann (PF) grades

Representative MRI images of patients with different Pfirrmann grades are reported in Fig. 1.

**Fig. 2:**
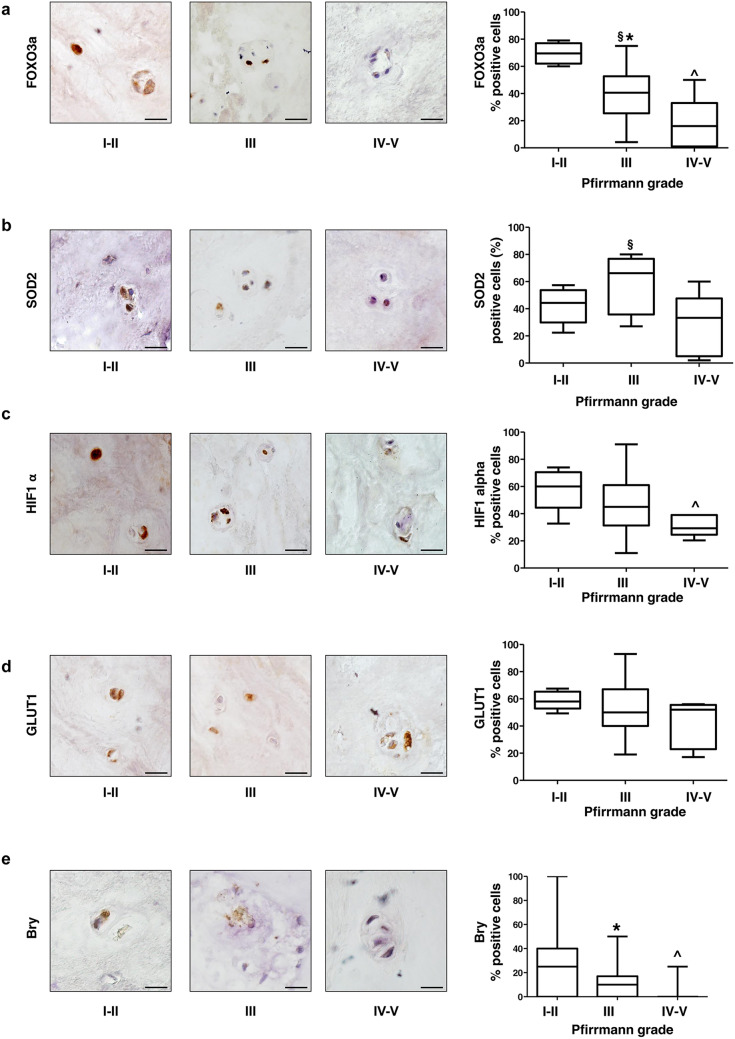
Detection of FOXO3a (**a**), SOD2 (**b**), HIF1α (**c**), GLUT1 (**d**), and Bry (**e**) expression by immunohistochemistry. Immunohistochemical analysis was performed on IVD tissues with different Pfirrmann grades of degeneration. Protein levels were quantified by densitometric analysis of immunostaining using ImageJ software and expressed as the percentage of positive cells per area (five sections per sample; Pfirrmann I–II group, *n* = 6; Pfirrmann III group, *n* = 40 and Pfirrmann IV–V group, *n* = 9). Results are reported as a whisker box plot representing the min to max (the line indicates median). *p < 0.01 (Pfirrmann III group vs. Pfirrmann I–II group); ^*p* < 0.01 (Pfirrmann IV–V group vs. Pfirrmann I–II group); ^§^*p* < 0.01 (Pfirrmann III group vs. Pfirrmann IV–V group). Scale bars 20 μm

To assess whether the study of key molecules could help to reveal a predisposition to IDD or potential functional recovery of the damaged IVD after surgery, we assessed the expression of specific proteins in relation to the patient parameters described above. We focused on proteins previously demonstrated, both in our studies and in the literature, to be involved in key signaling pathways for maintaining disc homeostasis (Penolazzi et al. 2018, 2019). These included FOXO3a, SOD2, HIF1α, GLUT1, and Bry, assessed by immunohistochemical analysis on IVD tissue samples from each enrolled patient (Fig. 2). FOXO3a, one of the best-known genes associated with human longevity, encodes a transcription factor that promotes cell protection and tissue maintenance, including the IVD (Alvarez-Garcia et al. 2017). Its protective effects are mainly due to the activation of its target SOD2 (superoxide dismutase type 2), an antioxidant enzyme that promotes the dismutation of superoxide into hydrogen peroxide (H_2_O_2_) (Alvarez-Garcia et al. 2018). This enzyme is critical for maintaining redox homeostasis in the IVD, preventing cellular senescence and ECM degradation (Wang et al. 2016). An interesting, yet complex, relationship involves FOXO3a and HIF1α. The transcription factor HIF1α is essential for the hypoxic physiological environment of the IVD and for maintaining disc health (Yang et al. 2023a, b). A feedback mechanism has been suggested in which FOXO3a influences HIF1α activity and HIF1α can induce FOXO3a expression, contributing to disc adaptation to hypoxia under different pathophysiological conditions. Furthermore, FOXO3a can negatively regulate HIF1α and its downstream targets, suggesting a role in promoting cell survival under conditions of severe hypoxic stress (Fasano et al. 2019). Consistent with these findings, our immunohistochemical experiments showed that, regardless of age and sex, low levels of FOXO3a, SOD2, and HIF1α expression were significantly associated with advanced disc degeneration (Pfirrmann group IV–V). A peculiarity concerns SOD2: unlike the two transcription factors, its expression was significantly increased in moderately degenerated discs (Pfirrmann III) compared to the Pfirrmann group I–II. This is consistent with recent data suggesting that SOD2 upregulation helps counteract reactive oxygen species (ROS) accumulation during moderate IDD (Tamagawa et al. 2024). However, when severe degeneration occurs, additional and partially understood molecular mechanisms, beyond the scope of this study, may lead to reduced enzyme activity and excessive ROS production, disrupting the redox imbalance and accelerating IDD progression (Li et al. 2022; Penolazzi et al. 2019). These findings highlight the importance of not limiting biomarker assessment to individual protein levels. Instead, a broader understanding of the interplay between various contributing factors is crucial to accurately define disease progression in IDD. Supporting this, we observed that GLUT1, a dominant glucose transporter in IVD and an important phenotypic marker of IVD cells in the hypoxic niche (Johnston et al. 2023), did not show significant changes across all samples, despite being a target of HIF1α.

As expected, Brachyury (Bry) expression decreased significantly with more advanced stages of disc degeneration (Fig. 2). This transcription factor Bry has a recognized protective role against disc degeneration (Wu et al. 2023). Its loss leads to metabolic dysregulation and reduced synthesis of key ECM components such as collagen II and aggrecan (Xia et al. 2024).

To further explore the relationships between these proteins and the parameters reported in Table 1, we conducted a cross-sectional analysis focusing on patients with Pfirrmann grade III (*n* = 40, 73%), the largest subgroup. This grade is most prevalent in patients under 60 years of age (Oh and Yoon 2017) and is particularly interesting for therapeutic development, given that degeneration at this stage is considered potentially reversible (Hickman et al. 2022). Expression levels of the analyzed proteins were compared between groups, and appropriate statistical analyses were performed (see Material and methods section). As expected, we observed a positive correlation between FOXO3a and SOD2 (*r* = 0.328, *p* < 0.05), HIF1α (*r* = 0.344, *p* < 0.005), and GLUT1 (*r* = 0.325, *p* < 0.05). A particularly strong positive correlation was observed between HIF1α and GLUT1 (*r* = 0.692, *p* < 0.001) (Table S1).

**Fig. 3:**
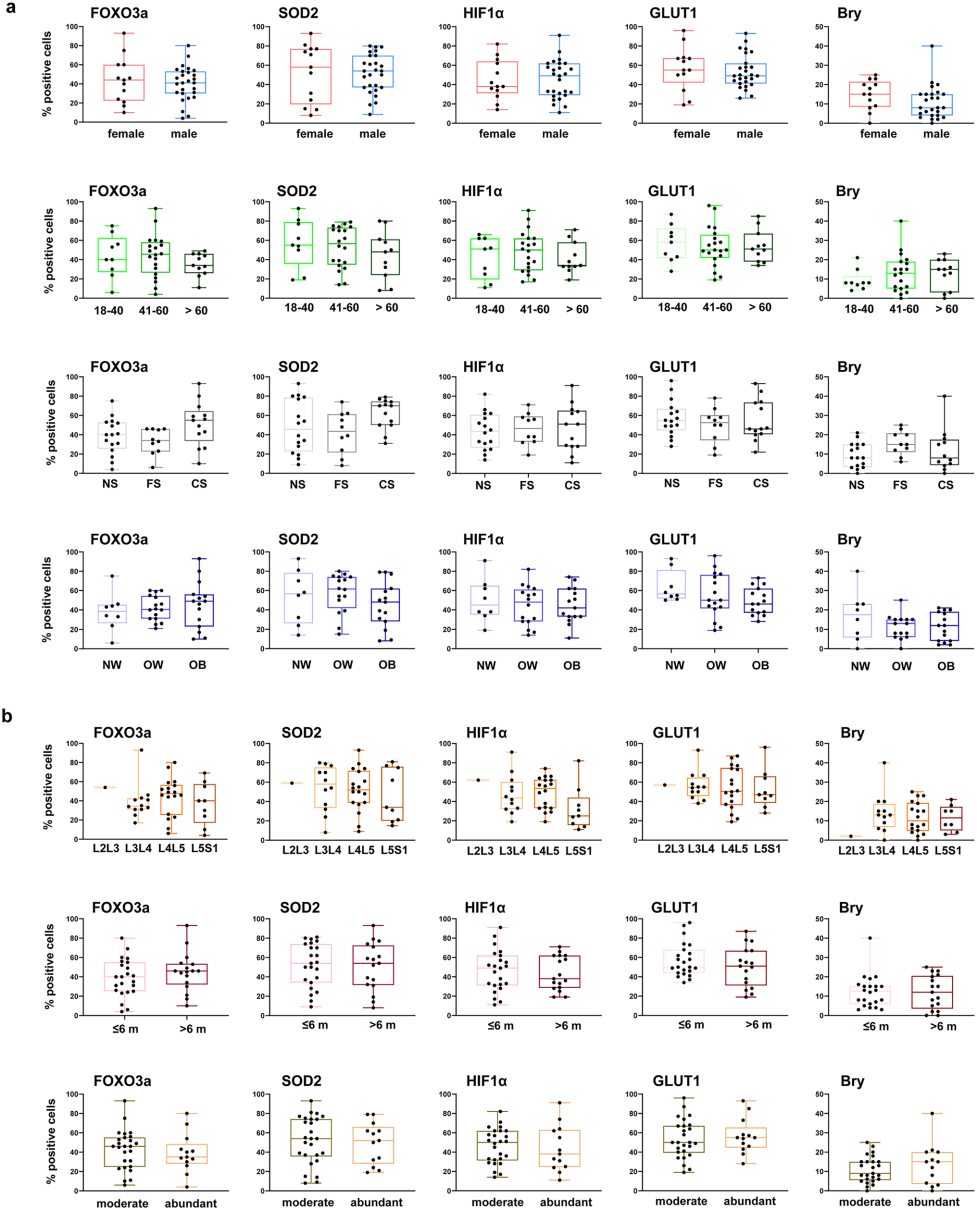
Analysis of FOXO3a, SOD2, HIF1α, GLUT1, and Bry expression in IVD tissues with Pfirrmann grade III. Protein levels were assessed by immunohistochemistry and quantified by densitometric analysis of immunostaining using ImageJ software. Results were expressed as the percentage of positive cells per area, based on five representative sections per sample (*n* = 40, Pfirrmann grade III). Data are presented as box-and-whisker plots, displaying min to max (the line indicates the median). Comparisons were made on the basis of the following parameters: **a** sex (female, male), age (18–40, 41–60, > 60 years), smoking status (non-smoker [NS], former smoker [FS], current smoker [CS]), and body mass index (BMI) (normal weight [NW], overweight [OW], obese [OB]); **b** anatomical site of surgery (L2–L3, L3–L4, L4–L5, L5–S1), duration of symptoms before surgery (< 6 months, ≥ 6 months), and area of infiltration (moderate, abundant)

In addition to the parameters reported in Table 1 (age, sex, smoke exposure, BMI, anatomical site of surgery, and duration of symptoms before surgery), patients were stratified according to the density of inflammatory cells/mm^2^ (moderate vs. abundant) in the biopsy. The results reported in Fig. 3 showed no statistically significant differences in protein expression between the different patient subgroups.

**Fig. 4:**
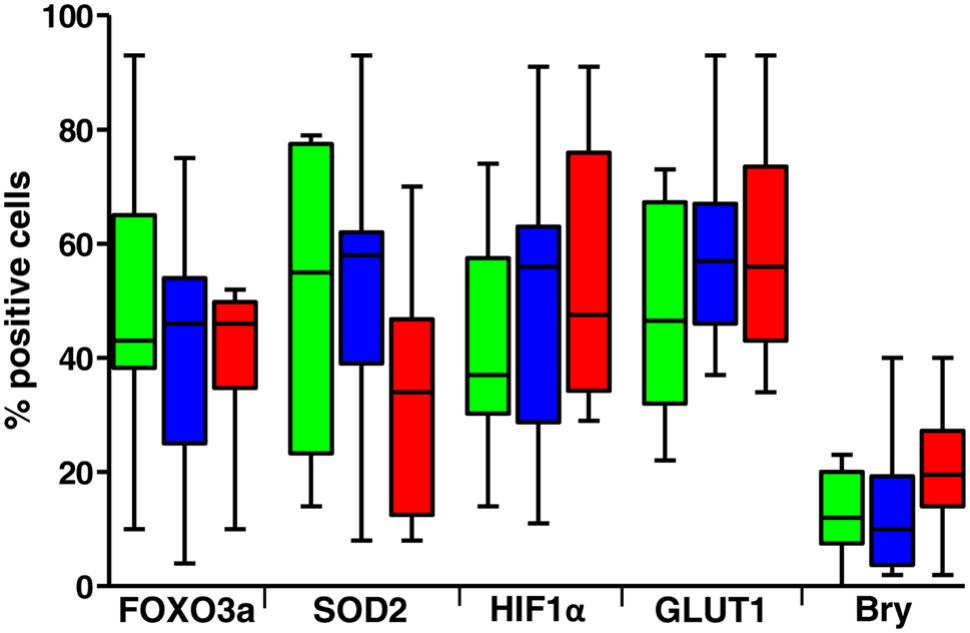
Correlation between FOXO3a, SOD2, HIF1α, GLUT1, and Bry expression levels and the onset of chronic pain or relapse. Results were expressed as percentage of positive cells per area, based on five representative sections per sample (*n* = 24, Pfirrmann grade III). Data are presented as box-and-whisker plots, displaying min to max (the line indicates the median). Patients without chronic pain after surgery (green), with joint inflammatory symptoms after several months (blue), and with short-term relapses (red)

Within the Pfirrmann grade III cohort, a subgroup of 24 patients provided follow-up data at 6 or 12 months after surgery. We examined whether the expression levels of FOXO3a, SOD2, HIF1α, GLUT1, and Bry were associated with postsurgical outcomes, such as chronic pain or relapse. Of these patients, 75% (18/24) confirmed complete healing after surgery. However, 62.5% (15/24) experienced joint inflammation within a few months, and 25% (6/24) developed short-term relapses. The aim was to understand whether a specific molecular signature related to these proteins could influence these postsurgical outcomes. Preliminary data, shown in Fig. 4, revealed some trends; however, no statistically significant differences were observed between the three groups. Certainly, enrolling a larger number of patients, comparing patients with different Pfirrmann grades, and examining additional molecular pathways could yield more informative and conclusive data. However, translating parameters such as perceived pain intensity and type into objective values remains a challenge. Despite the widespread use of standardized assessment tools, such as the Visual Analogue Scale (VAS), short- and medium-term pain outcomes remain controversial in clinical studies, especially considering the influence of occupational variables and psychological factors (Escalona-Marfil et al. 2020). In this regard, it has been reported that between 30% and 70% of patients experience persistent pain after surgery and fail to achieve satisfactory postoperative outcomes (Wang et al. 2022).

The findings presented highlight the complexity of identifying a reliable molecular signature for the onset of IDD or predicting postoperative recovery on the basis of patient characteristics and lifestyle. Even focusing on specific canonical markers within the IVD pathophysiological microenvironment remains a significant challenge. This difficulty should not discourage continued diagnostic and prognostic research in the field, as IVD degeneration stems from several underlying molecular and mechanical factors. This multifactorial nature of IDD indicates intricate biological phenomena exhibiting molecular redundancy, where multiple molecules perform similar functions to ensure cellular survival. Moreover, these molecules may initiate signaling pathways that are yet to be fully elucidated, but which hold promise for the identification of new diagnostic and therapeutic approaches.

## Supplementary Information

Below is the link to the electronic supplementary material.

Supplementary file1 (DOCX 13 KB)

## Data Availability

No datasets were generated or analysed during the current study.
