# Supplementary material for: Immunohistochemical analysis to detect a molecular signature in intervertebral disc degeneration
Source: Histochem Cell Biol. 2025 Nov 25;163(1):109. doi: 10.1007/s00418-025-02434-w (PMC12647181; doi:10.1007/s00418-025-02434-w)
Supplement: Supplementary file 1 — Supplementary file1 (DOCX 13 KB) [file 418_2025_2434_MOESM1_ESM.docx]

Title:

Immunohistochemical analysis to detect a molecular signature in intervertebral disc degeneration

Authors:

Letizia Penolazzi, Chiara Angelini, Riccardo Nadalini, Anna Chierici, Elisabetta Lambertini, Chiara Sief, Pasquale De Bonis, Roberta Piva

Journal:

Histochemistry and Cell biology

**Table S1.** Correlation coefficients (r) between the expression levels of the analyzed proteins in the PF III group.

|  | **FOXO3a** | **SOD2** | **HIF1α** | **GLUT1** | **Bry** |
| --- | --- | --- | --- | --- | --- |
| **FOXO3a** | — |  |  |  |  |
| **SOD2** | 0.328* | — |  |  |  |
| **HIF1α** | 0.344* | 0.107 | — |  |  |
| **GLUT1** | 0.325* | 0.223 | 0.692*** | — |  |
| **Bry** | -0.112 | -0.000 | 0.297 | 0.253 | — |

* p<0.05, ** p<0.01, ***p<0.001
